# Quality assessment of virus-like particle: A new transmission electron microscopy approach

**DOI:** 10.3389/fmolb.2022.975054

**Published:** 2022-11-25

**Authors:** Salomé De Sá Magalhães, Emiliana De Santis, Saba Hussein-Gore, Mathieu Colomb-Delsuc, Eli Keshavarz-Moore

**Affiliations:** ^1^ Department of Biochemical Engineering, University College London, UCL, London, United Kingdom; ^2^ National Physical Laboratory, NPL, Teddington, United Kingdom; ^3^ Vironova AB, Stockholm, Sweden

**Keywords:** TEM, vaccines, VLP, LMIC, SMEs, CQA, CNN

## Abstract

Transmission electron microscopy (TEM) is a gold standard analytical method for nanoparticle characterization and is playing a valuable role in virus-like particle (VLP) characterization extending to other biological entities such as viral vectors. A dedicated TEM facility is a challenge to both small and medium-sized enterprises (SMEs) and companies operating in low-and-middle income countries (LMICs) due to high start-up and running costs. A low-voltage TEM solution with assisted image acquisition and analysis such as the MiniTEM system, coupled with Vironova Imaging and Analysis Software (VIAS) could provide an affordable and practical alternative. The MiniTEM system has a small footprint and software that enables semi-automated data collection and image analysis workflows using built-in deep learning methods (convolutional neural networks) for automation in analysis, increasing speed of information processing and enabling scaling to larger datasets. In this perspective we outline the potential and challenges in the use of TEM as mainstream analytical tool in manufacturing settings. We highlight the rationale and preliminary findings from our proof-of-concept study aiming to develop a method to assess critical quality attributes (CQAs) of VLPs and facilitate adoption of TEM in manufacturing settings. In our study we explored all the steps, from sample preparation to data collection and analysis using synthetic VLPs as model systems. The applicability of the method in product development was verified at pilot-scale during the technology transfer of dengue VLPs development from a university setting to an LMIC- based vaccine manufacturing company, demonstrating the applicability of this analytical technique to VLP vaccine characterization.

## Introduction

Virus like particles (VLPs) are made of multiple copies of the same or different peptidic building blocks (monomers) which self-assemble into higher order nanostructures that mimic the morphology and size of viruses but lack the genetic material and therefore are non-infectious (Comas-Garcia et al., 2020). The size of VLPs typically ranges from 20 nm to 200 nm and can be manufactured both recombinantly and synthetically. Despite the challenges in the scale up of VLPs manufacturing, as well as in the characterization of their complex assemblies, these particles have become powerful tools in vaccinology and biomedical research (Qian et al., 2020). For example, VLPs find application as delivery vehicles (DNA encapsulation); antigen presentation (adjuvant) and as vaccine platform (e.g., GARDASIL^®^, against human papilloma virus, Engerix B^®^, against hepatitis B virus, Epaxal^®^, against hepatitis A virus) (Tariq et al., 2022).

Recombinant VLPs can be produced in a variety of systems, from bacteria to mammalian cells, allowing flexibility in the choice of expression platforms (Fuenmayor et al., 2017). Synthetic VLPs are produced *via* chemical synthesis and can be inspired by nature (Castelletto et al., 2016), mimicking existing bioactive sequences, or be designed *de novo* (Noble et al., 2016; De Santis et al., 2017; Kepiro et al., 2020). They offer high control over their sequence, structure and assembly, as intra- and intermolecular interactions are engineered by design. This is advantageous and makes them ideal candidates in the development of model systems and reference materials (Briones et al., 2022) to benchmark critical quality attributes (CQAs) of VLP-based vaccines and drugs, during development and manufacturing. Additionally, VLPs can be engineered to display multiple biological activities, concomitantly (Castelletto et al., 2016), and can span sizes from 10 nm to few microns (Noble et al., 2016).

## Analytics for quality attribute assessment

In vaccine production, bioactivity, potency, and stability of VLPs are crucial to ensure an active and safe outcome. Due to the structural complexity of VLPs, bioactivity depends not only on the properties of the building blocks (monomers), but crucially, also on their correct assembly and overall VLP morphology, which must be ensured throughout the development and manufacturing cycles. In essence, the bioactivity for VLPs is more than the sum of its parts. Correct morphology (shape) and size are CQA to ensure functional VLPs and specific measurands (i.e., the quantity to be measured) must be selected to describe these.

The structural complexity of VLPs, alongside the lack of clear acceptance criteria for the VLPs CQAs, makes manufacturing and characterization of VLPs challenging. Suitable analytical tools, methods and standards must be available for their assessment. A broad variety of analytical tools are available for biochemical, biophysical, functionality and stability characterization (Kumar et al., 2020; Nooraei et al., 2021). The assessment of CQA of VLP monomers (e.g., chemical identity and purity) can be informed by techniques and methods employed in the quality control of small molecules and biologics, which are already well-embedded in manufacturing settings. However, CQA associated with the final nanostructures (e.g., morphology, size, polydispersity) are harder to evaluate for biological nanoparticles, such as VLPs and viral vectors, where most of the analytical tools, methods, and standards to assess CQA of nanoparticles are typically optimized for inorganic nanoparticles and/or are not embedded in manufacturing settings. Whilst several techniques can provide information on size and polydispersity of VLPs, these can fail to provide information on their morphology, structural integrity, and any variations to these, which are often at the nanoscale and beyond the detection limit of most techniques commonly employed in manufacturing settings.

Amongst the techniques available, transmission electron microscopy (TEM), provides high resolution information at the nanoscale, playing a key role in defining quality control criteria during process development and manufacturing (De Santis and Ryadnov, 2021). Concomitant information on size, polydispersity, morphology, and structural integrity, can be obtained by TEM at nanoscale resolution, thus making the technique an invaluable tool for the characterization of viral and VLP-based vaccines. However, the use of TEM as routine analytical tool in manufacturing settings is hampered by several factors, including: 1) complexity of sample preparation, typically employing radioactive stains, 2) need for specialist facilities and highly trained staff leading to high costs 3) lack of validated methods and reliable reference materials for biological nanoparticles, such as VLPs. These barriers are even harder roadblocks for SME operators and manufacturers operating in low and middle-income countries (LMIC). In LMICs the frequent outbreaks of infectious diseases, coupled with limited resources (Plotkin et al., 2017; Kis et al., 2019; Khan et al., 2021), hamper vaccine development. Bringing in a user-friendly, accurate and precise method for nanoparticle characterization can significantly improve labor and cost efficiency for VLP vaccine development.

Low voltage TEM systems, such as the MiniTEM (Vironova AB), could help address some of these limitations, thus enabling the use of TEM as routine analytical tool in vaccine development. The reduced installation and maintenance costs of low-voltage TEM, due to the small footprint and ease of use, makes it compatible with any room or laboratory settings and easy to integrate within existing analytical pipelines. The MiniTEM system’s resolution of 1 nm allows nanoparticles in the size-range of 10 nm–500 nm to be observed, thus enabling the analysis and characterization of therapeutic nanoparticle delivery systems used in vaccines, gene therapy and drug delivery. The MiniTEM system is operated *via* a user-friendly software with automated features, VIAS, used to control the microscope, visualize and image samples as well as analyze data for users with little previous knowledge including automated microscope alignment and autofocus features. The software encompasses pattern recognition, AI and machine learning capabilities that enable it to perform advanced particle characterization, classification and measurements. VIAS uses a networked SQL server for optimal performance for data sharing and handling large data sets and storage capabilities.

The MiniTEM system is therefore tailored for non-experts in TEM to rapidly obtain meaningful particle characterization data that complements existing routine analytics in process development, thereby reducing the dependence on expert microscopists and subjective analysis, lifting some of the obstacles that have held back the routine use of TEM analysis in vaccine development settings (Verleysen et al., 2019).

Availability of additional information and more applicable analytical tools and methods for the biophysical characterization of VLPs would positively affect the rate and speed of approval of this class of vaccines in commercial applications and allow the industry to comply with the strict and increasing regulatory demands (Plotkin et al., 2017). To facilitate the uptake of TEM in process development and manufacturing settings, we generated a conceptual framework of the challenges for effective implementation of specific CQA analysis of VLPs using the MiniTEM system. Firstly, the development of a TEM analytical pipeline for VLPs, from sample preparation to data collection and analysis, was optimized to minimize the use of specialist infrastructure and training. Secondly, a proof of concept demonstrating the applicability of the method and analysis workflow to a vaccine product under development was conducted. The MiniTEM system (MiniTEM electron microscope + VIAS software) is currently being designed and developed to meet the requirements of quality control (QC) environments where the setup provides a cohesive solution for safety in data handling and traceability, hence making it a system of choice to be used in a quality-controlled environment and workflow. The VIAS software as such could be used with other TEM systems, but further developments to ensure a QC through the whole workflow would be required to ensure the data and metadata traceability and integrity conservation throughout the system transfers that such a workflow would involve.

The study design and method development focused on measurands that provided quantitative information that describe CQA of VLPs. Specifically, we developed a TEM method for the morphological (roundness and aspect ratio) and size (Feret diameter and area) characterization. The overall study design aimed at assessing the intra-laboratory and inter-laboratory reproducibility for the TEM analysis of selected VLP model systems for further method development.

## TEM workflow for the analysis of VLPs

### Optimization of specimen preparation using non-radioactive stains

**FIGURE 1:**
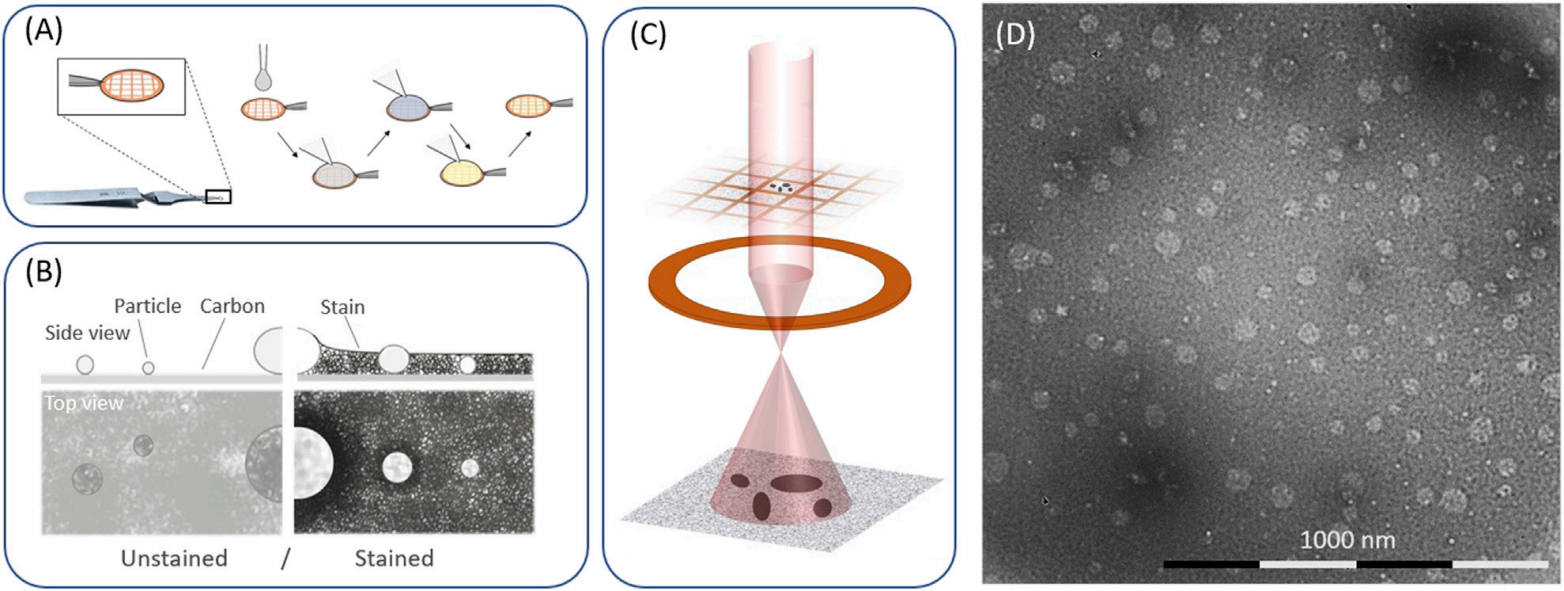
TEM workflow. **(A)** Depiction of an EM grid, used as support, and representative steps of a specimen preparation with specimen deposition (1), washing (2, 3), staining (4) and drying (5). **(B)** Schematic representations of an unstained and stain specimen. **(C)** image formation in an electron microscope. **(D)** representative image of a VLP prepared and imaged by negative stain TEM.

Preparation of efficient embedding and even specimen spreading on the grid support is a prerequisite for an unbiased TEM assessment and imaging (Figure 1A) (Hauser et al., 2020). The stain helps to maintain the specimen integrity while ensuring a good contrast (Figure 1B) for biological samples. Indeed, the image formation in TEM results from the formation of a magnified image of the transmitted electrons from an electron beam passing through a specimen (Figure 1C). A few microliters of sample are deposited onto a TEM grid. Due to the inherently low contrast of biological samples under the electron beam, heavy metals are typically used to increase sample contrast by staining either the sample (positive staining) or the background (negative staining) (Brenner and Horne, 1959; Unwin, 1974). The contrast originates in part from the electron density of the specimen, heavy metals provide a good contrasting agent, with uranyl acetate (UA) being the most widely used stain for imaging biological samples. However, the inherent radioactivity of uranyl acetate and the consequent need for licensed facilities and specialist training, for handling and disposal limits the technological transfer of these methods to manufacturing settings. In this context, alternative solutions, such as employing non-radioactive staining reagents are important to address and promote adoption of TEM in SMEs and LMICs and has the potential to significantly accelerate routes to market for VLP-based vaccines. Significant efforts are ongoing to develop effective, safe, and stable non-radioactive stains and a range of commercially available non-radioactive stains are today available with different pH, metal content or concentrations depending on the analytical needs and specimen properties. However, a limited number of protocols optimised for the TEM analysis of VLPs particles using non-radioactive stains are available, hence limiting their widespread use for this application.

Additionally, vaccines developed during manufacturing often have a complex formulation to ensure stability of the VLPs and these may hamper the preparation of TEM specimens, causing for example stain precipitation and variable staining across the grid. It is therefore essential to take the potentially complex composition in mind when developing protocols for sample preparation. To address this complexity, we have employed Dengue VLPs (See Supplementary information). Evaluating these particles using TEM is an increasing part of defining QC criteria during development and ultimately manufacturing. The quality assessment of these particles is a critical step for quick decision making, saving time, and consequently, costs. We have explored the use of Nano-W™ and NanoVan™ in the preparation of VLP specimens for TEM analysis and demonstrated the equivalence to uranyl acetate. Specimen preparation protocols were developed to optimize stain thickness, sample, and stain interaction (e.g., sample status and aggregation due to precipitation) and grid quality were evaluated enabling to fine tune the sample/grid preparation process. Through this optimized protocol we have reached equivalence of staining using radioactive (uranyl acetate) and non-radioactive stains (Nano-W™) providing greater flexibility for VLP sample preparation (See Supplementary information).

The availability of well-established sample preparation protocols is equally crucial. The nature of the specimen preparation, which requires several steps (e.g., sample deposition, blotting, and stain) and iterations, often suffers from poor reproducibility which can affect the accuracy of the analysis. The experience of the operator also plays a critical role in determining the final quality of the specimens. As a result, sample preparation is widely recognized as the bottleneck of TEM analysis and several efforts are ongoing to develop methods for semi-automated sample preparation to minimize manual handling of the samples, thus increasing safety, throughput and reducing variability (Benmeradi et al., 2015; Monninger et al., 2016; Strader et al., 2018).

### Optimization of data collection (MiniTEM) and data analysis using artificial intelligence and neural network

Establishing a data collection scheme for a prepared TEM grid is key to ensure that the data is of suitable quality and statistically representative of the sample. TEM currently offers structural information at very high resolution, often at the expense of the total number of particles that can be investigated. It is therefore essential to devise a data collection schemes that can balance the need to obtain a data set which is representative of the bulk of the samples, without losing the high-resolution information that can accurately describe the measurands of interest. In practice, this is often a compromise between the resolution and the field of view (FOV) to obtain statistically significant information. Figure 1D specific guidance is available to support the users in selecting the most appropriate data collection scheme with respect to the resolution, field of view and number of representative particles (International Organization for Standardization, 2020). The size of the particles under investigation dictates the most appropriate data collection scheme.

Synthetic VLPs, are ideal model systems for method development, due to the rationally engineered interactions which offer high control over their self-assembly and morphology (De Santis and Ryadnov, 2015). Synthetic VLPs also represent ideal candidate reference materials for applications in vaccine development and gene delivery (Briones et al., 2022). Whilst development of VLPs was out of the scope of this study, a specific system was employed as a test model in this proof of concept to support the method development, focusing on key morphological parameters. In our proof of concept, we optimized the data collection scheme, e.g., resolution (0.7 nm/px) and field of view (1,500 nm × 1,500 nm) to obtain information on the key measurands that described the size (Feret diameter and area) and shape (roundness and aspect ratio) for synthetic VLPs. To enable technical transfer of the method beyond its original application, we selected VLPs with a size of around 20 nm, which is comparable to that of some of the viral vectors (such as AAV) typically employed in vaccines development or as gene delivery vectors.

Automated or semi-automated data collection is particularly crucial to minimise/remove bias from the operator during data collection. We employed VIAS software for automated data collection to image a minimum of 3 independent areas well-spaced across the TEM grid to obtain a statistically relevant amount of detected VLPs particles (See Supplementary Information).

Data analysis is employed to derive the numerical values of the selected key measurands. Several approaches are available for data analysis of TEM images, such as ImageJ (Schindelin et al., 2012; Schneider et al., 2012). Although well established, these data analysis methods can suffer from operator bias, are often time consuming and require significant user-input and specialist skills, which limit the uptake of TEM as mainstream analytics in fast-paced manufacturing settings.

Significant efforts over the past few years have been dedicated to the development of artificial intelligence (AI) approaches for the analysis of TEM images, hence speeding-up processes by reducing operator input and improving the objectivity, precision, reliability, and robustness of the obtained data by removing user bias. Those advantages make these approaches good candidates to be implemented in a quality-controlled framework. Recent developments and implementation of analytical workflows using Convolutional Neural Networks (CNN) applied to image analysis of biological specimen appear promising. In this approach, the network is fed with a manually annotated set of data, considered as the raw data, and used to train the CNN model to develop and tune algorithms able to independently perform image segmentation, particle detection and classification on a new set of images. Using this approach with the VIAS software CNN training module, synthetic VLP particles were manually annotated to inform and train the model to enable discrimination between VLP particles, background, and debris. After screening the specimen for determining areas suitable for imaging and subsequently choosing the most appropriate field of view, a set of references images was acquired and annotated to be used as ground truth for the generation and training of the CNN model, prior to testing on a validation data set.

The model was subsequently applied to new TEM images and the automated particle detection performance examined and assessed. The data collection and the analysis workflows generated from these samples were used for the development of different CNN models, to enable automated VLP particle detection and measurement of quality attributes, such as diameter and roundness. The analysis using the CNN model was significantly faster and required no manual input, allowing for a shorter turnaround with less user-variability.

**FIGURE 2:**
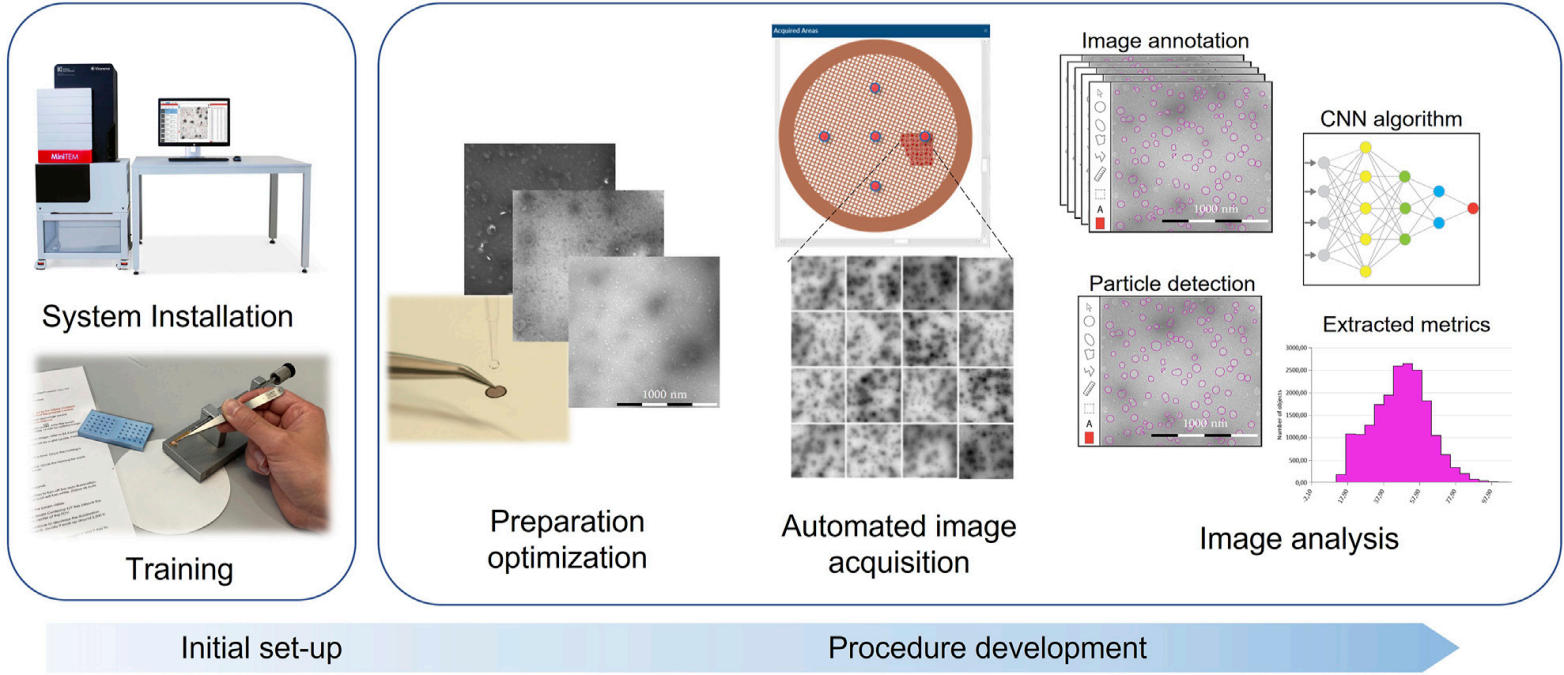
Development process for implementation of TEM workflow. After an initial set-up consisting of the system installation and personnel training, the preparation method is optimized by testing different stains, supports and preparation conditions. The prepared specimens are then imaged using an automated data acquisition to screen several grid areas and ensure a good representativity of the sample. Finally, the acquired images are fed into the CNN algorithm, trained *via* manual image annotation prior to the automated analysis, the resulting detection can be visually evaluated, and the data used for specimen evaluation. This whole process can be implemented into a validation framework.

In this study, the CNN models successfully detected VLPs representing diverse morphologies under varied staining thicknesses. Moreover, to demonstrate the applicability of the method and analysis workflow, the models were successfully applied to Dengue VLP vaccine candidates, providing a flexible and tunable model for future studies and analyses (Figure 2). This has been a successful first step proof of concept for using MiniTEM. Further and future work will focus on assessing other critical quality attributes, such as sample purity, different morphologies and sizes (See Supplementary Information).

The images were analyzed using the MiniTEM proprietary software VIAS, where equivalence of staining using radioactive and non-radioactive stains with adapted/optimized protocols was demonstrated. For both cases, attributes such as size and roundness showed acceptable/good correlation (data not shown). Through image data collected during the study, a dedicated CNN (ref) model was created by Vironova to enable automated VLP particle detection and measurement of quality attributes that include particle count, diameter, and roundness. The CNN model is part of a simple workflow that requires minimal training to operate and has the potential to accelerate decision-making processing during VLP vaccine development (Figure 2). The use of AI and CNN in vaccine development is showing potential on particles such as analysis of extracellular vesicles, exosomes, lentiviral vectors, and other enveloped, pleomorphic particles that are traditionally more difficult for image analysis classification in part due to their variable size and shape.

## Final remarks

In this perspective we have provided an overview of the limiting factors in the application of TEM in development and manufacturing settings, highlighting key challenges within a typical TEM workflow. Our proof-of-concept study aims at providing solutions to enable a wider uptake of TEM, with emphasis on facilitations for LMIC countries, where a TEM analytical workflow was created towards the development of an optimized method for the assessment of CQAs of VLPs with defined acceptance criteria. The applicability of the method and analysis workflow to a Dengue VLP vaccine product under development by PT Bio Farma and UCL, was demonstrated, providing technical solutions for vaccine development by LMICs to tackle endemic diseases.

An emphasis on simplification and timesaving was a central point in the *in-situ* applicability of an alternative user-friendly visual technology to standard TEM. This also follows the trend of using AI and machine learning to alleviate and simplify processes by allowing smart automation and introducing non-subjectivity, an increasing requirement from regulatory authorities.

By addressing the obstacle steps of the workflow, from sample preparation to data collection and analysis we strove to enable an easy technical transfer into process development and manufacturing settings and uptake by non-expert microscopists.

Having demonstrated that the MiniTEM is a suitable tool for quality control in vaccine development, further studies will aim at providing detailed analysis of a range of VLPs and related self-assembles systems to support the applicability of MiniTEM and its software to an industrial setting.

This study is closely aligned with the aims of organizations and initiatives established in the UK to support medicine manufacture such as the UK Medicines Manufacturing Industry Partnership (MMIP), the vaccine manufacturing innovation center (VMIC), the Medicines Manufacturing Innovation Centre (MMIC), and academic/industry centers such as Vax Hub which promote efforts to accelerate time to market technology of advanced therapies for manufacturing and regulatory environments.

## Data Availability

The datasets generated for this study are available on request to the corresponding authors.
