# Supplementary material for: Quality assessment of virus-like particle: A new transmission electron microscopy approach
Source: Front Mol Biosci. 2022 Nov 25;9:975054. doi: 10.3389/fmolb.2022.975054 (PMC9732438; doi:10.3389/fmolb.2022.975054)
Supplement: Supplementary file 1 [file Table1.DOCX]

**Supplementary information** – de Sá Magalhães et al., Quality assessment of Virus-Like Particle: A new Transmission Electron Microscopy Approach

**Dengue VLP**

**VLP sample preparation and optimisation using non-radioactive stains:**

Sample grid preparation is a key prerequisite for TEM analysis, and the quality of the preparation is critical to automate image analysis and particle characterisation. The optimisation of non-radioactive stains is critical for adoption within SMEs and LMICs as such stains do not require licensed facilities (for radioactive stains) and thus has a potential a significant impact on accelerating routes to market for VLP-based vaccines.

**VLP Imaging:**

Imaging of the dengue VLPs prepared with radioactive (Uranyl Acetate) and non-radioactive (NanoVan & NanoW) stains was performed and examined. Based on the achieved results (**Figure 3**), we demonstrated equivalence of staining using radioactive and non-radioactive stains with adapted/optimised protocols. This provides greater flexibility for sample preparation since staining can be performed in a standard laboratory setting and negates the requirement for a licensed facility and increases the potential adoption and implementation within LMICs.

**Convolutional Neural Network (CNN) Model Creation:**

Through the image data collected during the study a dedicated CNN model was created by Vironova to enable automated VLP particle detection and measurement of quality attributes that include particle count, diameter, and roundness amongst others. The CNN model is housed within a simple workflow that requires minimal training to operate and has the potential to accelerate decision making processing during VLP vaccine development.

**Figure 3.** Demonstration of the equivalence of staining using radioactive (left-hand-side) and non-radioactive (middle and right-hand-side) stains with adapted/optimised protocols.

**Developing the Method**

Synthetic virus-like particles (VLPs) engineered at the National Physical Laboratory (NPL) were employed as candidate test and reference materials for method development for TEM. The preparation of the TEM grids was optimised to use non-radioactive staining (NanoW, NanoVan) and informed by the results for dengue VLP (Figure 4).


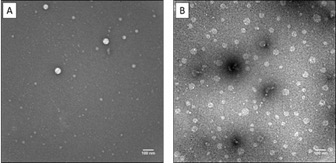


**Figure 4.** Representative images of virus-like particles (VLPs) stained with uranyl acetate (A) and NanoW (B), collected at 1500 nm FOV using the MiniTEM system and VIAS software.

**VLP CNN Model Creation**

Through the VIAS CNN training module, synthetic VLP particles were manually annotated to inform and train the model to enable differentiation between VLP particles, background, and debris. Each CNN model generated was trained on a minimum of 30 images with 100 VLP particle annotations and completed at least 100 iterations on the training data prior to testing on a validation data set (Figure 5)


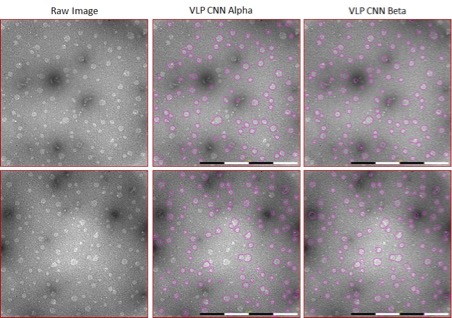


**Figure 5.** Representative TEM images of synthetic VLPs stained with NanoW (left column). VLP particle detection using the CNN model Alpha (1st iteration; middle column). Improved VLP particle detection using the CNN model Beta (2nd iteration; right column).

**Generation of adaptable CNN models for analysis of dengue VLPs**

The CNN models generated from this study successfully detected VLPs presenting diverse morphologies under varied staining thicknesses. The early resources invested in model training translated into a more efficient process to adapt the model to detect new VLP types such as the dengue VLPs that were part of the pilot technology transfer of study and provided a flexible and tunable model for future studies and analyses (Figure 6).


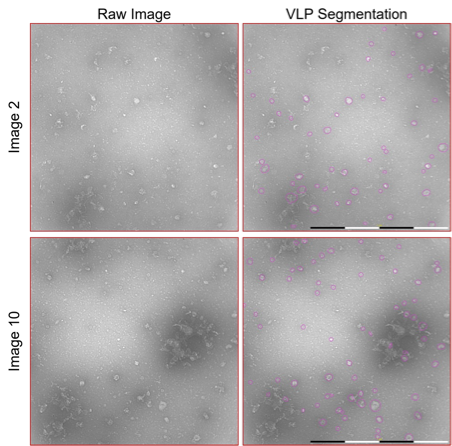


**Figure 6.** Representative images of DEN1DEN2 VLPs stained with NanoW. Raw images shown on the left with NanoW_VLP_v3 CNN particle detection on the right.

**Method applied to the data collection and analysis of dengue VLP**

The method validation procedure developed from this study was applied to the data collection and analysis of dengue VLP samples (Figure 7).


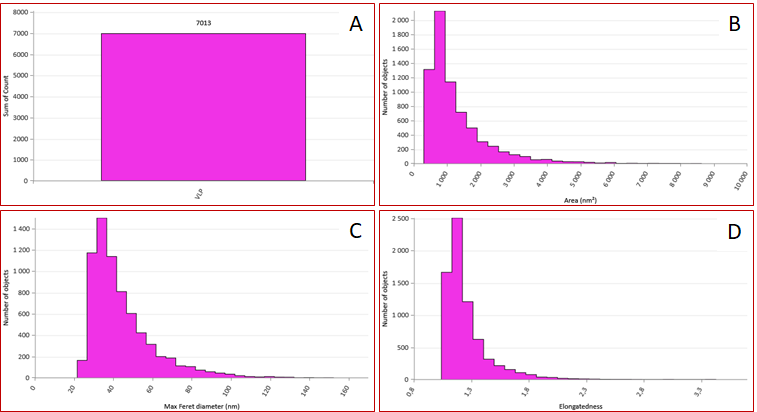


**Figure 7.** VIAS plots following NanoW_VLP_v3 CNN particle detection. Dengue VLP Particle Count (A), Area (B), Max Feret diameter (C) and Elongatness (D). The particles measured the predicted size 30-40 nm and displayed elongatedness properties that indicated the dengue VLPs were of suitable conformation and that the data correlated to the results from orthogonal methods such as TEM (data not shown).

This technique provides advantages over some of the existing approaches because, firstly the footprint is small, secondly there is no need to use radioactive material for sample preparation and thirdly ability to connect to an imaging software that can be used to analyse the morphological results quickly and ( if enough data taken) with statistical significance .

Further study with other VLPs and direct comparison with a traditional TEM system will enable this proof-of-concept to be fully realised for validation purposes.
